# Supplementary material for: Discovery of iridoid cyclase completes the iridoid pathway in asterids
Source: Nat Plants. 2025 Oct 3;11(11):2204–16. doi: 10.1038/s41477-025-02122-6 (PMC12626888; doi:10.1038/s41477-025-02122-6)
Supplement: Supplementary file 1 — Supplementary Figs. 1–15. [file 41477_2025_2122_MOESM1_ESM.pdf]

nature plants

Supplementary information https://doi.org/10.1038/s41477-025-02122-6

Discovery of iridoid cyclase completes the iridoid pathway in asterids

In the format provided by the authors and unedited

Table of contents

Supplementary Figures **2**

Supplementary Fig. 1. Secoiridoid pathway in different asterid orders 2

Supplementary Fig. 2. Photos of plants sampled for genome sequencing, tissue specific RNA-sequencing and single nuclei RNA-sequencing. 3

Supplementary Fig. 3. Identification of orthologs of known *C. roseus* secoiridoid pathway genes in *C. ipecacuanha* and *A. salviifolium* and tissue-specific expression data. 4

Supplementary Fig. 4. Single nuclei RNA-seq *C. ipecacuanha* young leaves. 5

Supplementary Fig. 5. Expression of iridoid and ipecac alkaloid pathway genes in single cell clusters. 7

Supplementary Fig. 6. Maximum-likelihood phylogenetic tree of ICYC amino acid sequences. 8

Supplementary Fig. 7. ICYC orthologs from various Asterid orders enable loganic acid biosynthesis in *N. benthamiana* 9

Supplementary Fig. 8. Reconstitution of the secoiridoid pathway of *C. ipecacuanha* and *A. salviifolium* in *N. benthamiana*. 10

Supplementary Fig. 9. Side products formed by CrISY and AmISY in the absence of cyclase 11

Supplementary Fig. 10. Electron ionization (EI) spectra of nepetalactol standard and enzymatic products. 12

Supplementary Fig. 11. ICYC activity with 8-oxocitronellal under tautomerization inducing conditions. 13

Supplementary Fig. 12. Maximum likelihood tree of ICYC and methylesterase amino acid sequences. 14

Supplementary Fig. 13. Structural overlay of CiICYC model with RsPNAE structure 15

Supplementary Fig. 14. Docking of nepetalactol stereoisomers to CiICYC and AmICYC models 16

Supplementary Fig. 15. Possible cyclization mechanisms. 17

Supplementary references **18**

Supplementary Figures

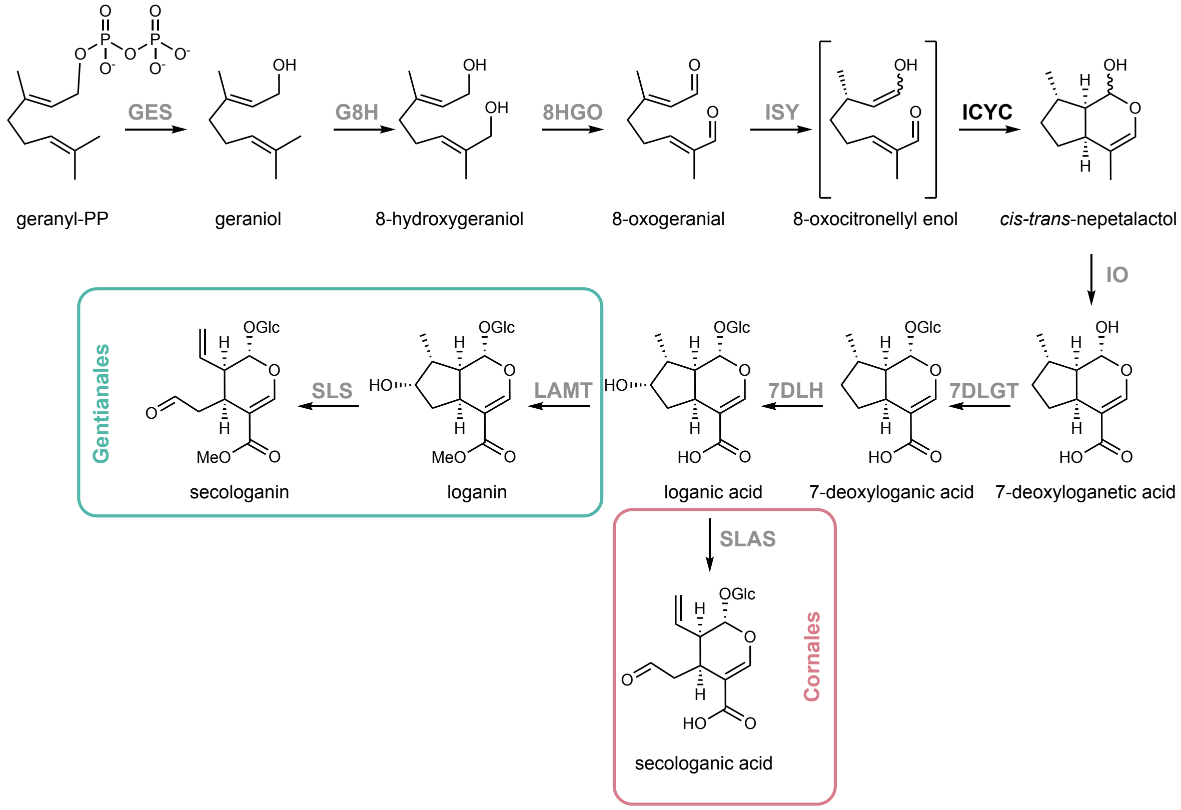

Supplementary Fig. 1. Secoiridoid pathway in different asterid orders. Complete secoiridoid pathway. Enzymes labeled in gray have been previously identified in other studies on *Catharanthus roseus* (Gentianales) and/or *Camptotheca acuminata* (Cornales). Generally, in the Gentianales (and other orders) the pathway end product is the methylester secologanin whereas in the Cornales lineage it is secologanic acid due to the absence of LAMT [1,2]. SLS and SLAS are orthologs and can both accept loganic acid as substrates [3]. GES, geraniol synthase; G8H, geraniol 8-hydroxylase; 8HGO, 8-hydroxygeraniol oxidase; ISY, iridoid synthase; ICYC, iridoid cyclase; IO, iridoid oxidase; 7DLGT, 7-deoxyloganetic acid glucosidase; 7DLH, 7-deoxyloganic acid hydroxylase; SLAS, secologanic acid synthase; LAMT, loganic acid methyltransferase; SLS, secologanin synthase.

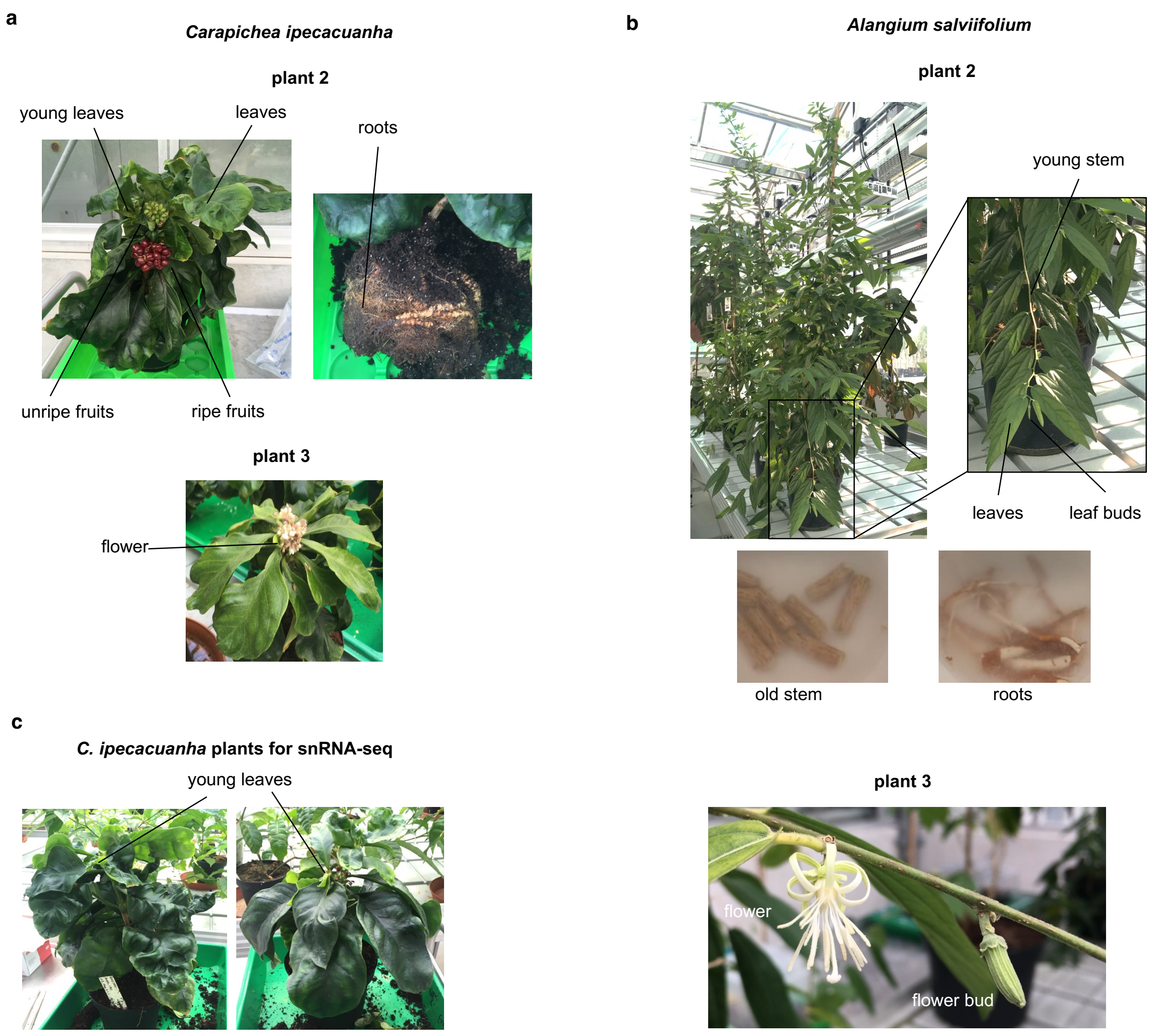

Supplementary Fig. 2. Photos of plants sampled for genome sequencing, tissue specific RNA-sequencing and single nuclei RNA-sequencing. a, *Carapichea ipecacuanha* “plant 2” and “plant 3” were 1.5 years old at the time of harvesting. **b**, *Alangium salviifolium* “plant 2” and “plant 3” were 2.5 and 4 years old, respectively at the time of harvesting. **c**, *C. ipecacuanha* young leaves for single nuclei RNA-seq (snRNA-seq) were harvested from 1.5-year-old plants. All plants were grown in a greenhouse with controlled conditions (12/12 hours light/dark 28-30°C/24-26°C, 70-80% humidity). Sequencing data for plants labelled as “plant 1” in this study were previously published [2] (https://www.ncbi.nlm.nih.gov/bioproject/PRJNA1169657).

a

enzyme name	short name	Accession *C. roseus*	Best blast hit *C. ipecacuanha*	% identity	Best blast hit *A. salviifolium*	% identity
geraniol synthase	GES	JN882024	Caipe.S184100	79	Alsal.S224590	66.5
geraniol 8-hydroxylase	G8H	KF561461	Caipe.S125190	81	Alsal.S141850	74.2
8-hydroxygeraniol oxidase	8HGO	KF302069	Caipe.S388690	87.3	Alsal.S112090	70.1
iridoid synthase	ISY	KJ873886	Caipe.S317540	77.9	Alsal.S308160	61.2
iridoid oxidase	IO	KF591593	Caipe.S160820	89.9	Alsal.S107160	77.8
7-deoxyloganetic glycosyltransferase	7DLGT	KF302067	Caipe.S283170	67.1	Alsal.S289890	66
7-deoxyloganic acid hydroxylase	7DLH	AB733667	Caipe.S364920	84.6	Alsal.S217620	72.8
loganic acid methyltransferase	LAMT	KF415116	Caipe.S248290	79.6	Alsal.S158890*	53
secologanin synthase	SLS	KM524262	Caipe.S229390	76.1	Alsal.S289930	64.3

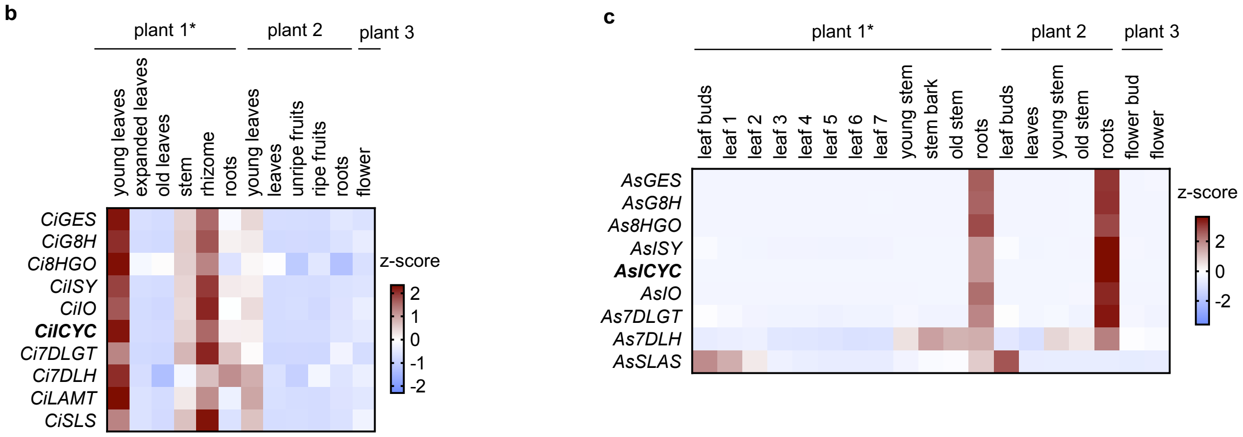

Supplementary Fig. 3. Identification of orthologs of known *C. roseus* secoiridoid pathway genes in *C. ipecacuanha* and *A. salviifolium* and tissue-specific expression data. a, Amino acid sequences of the previously published *C. roseus* pathway enzymes were subjected to BLAST (tblastn) against *C. ipecacuanha* and *A. salviifolium* transcripts (working gene models) generated from the annotated genomes. The hits with the highest sequence identities were considered orthologs and the activities of these gene products were confirmed in this study. Note, that for LAMT the hit with the highest sequencing identity in *A. salviifolium* (marked with an asterisk) showed comparably lower sequence identity. **b,** Tissue-specific expression analysis of identified iridoid pathway gene orthologs and the newly identified *CiICYC* in *C. ipecacuanha* shows high co-expression of identified iridoid pathway genes ortholog alongside the newly identified *CiICYC* in young leaves and rhizome. **c,** Tissue-specific expression analysis of identified iridoid pathway gene orthologs and the newly identified *AsICYC* in *A. salviifolium* reveals almost exclusive expression in the roots, apart from *AsSLAS*, which is also expressed in leaf buds and young leaves.

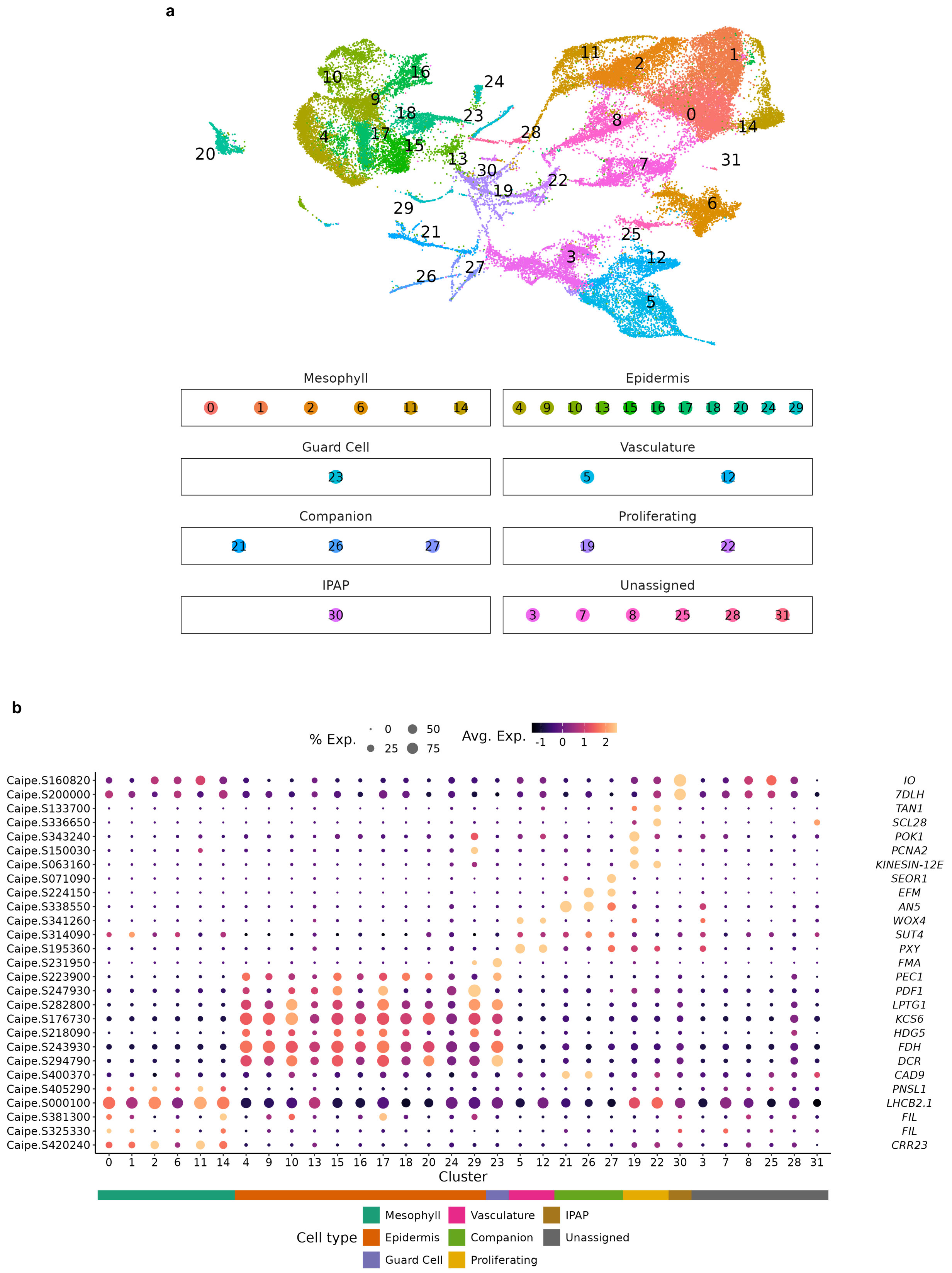

Supplementary Fig. 4. Single nuclei RNA-seq *C. ipecacuanha* young leaves. a, UMAP of average gene expression of two biological replicates of *C. ipecacuanha* young leaves ($n = 23{,}702$ cells for replicate 1, and 20,299 cells for replicate 2). Cell clusters were annotated as cell types using marker genes shown in **b**. IPAP, Internal Phloem Associated Parenchyma. **b**, Single-cell gene expression dot plot heatmap showing expression of orthologs of previously published marker genes for different cell types in other species (Supplementary Table 7). Color scale shows the average scaled expression of

each gene over the different cell clusters. Dot sizes indicate the fraction of cells of each cell cluster in which a given gene is expressed.

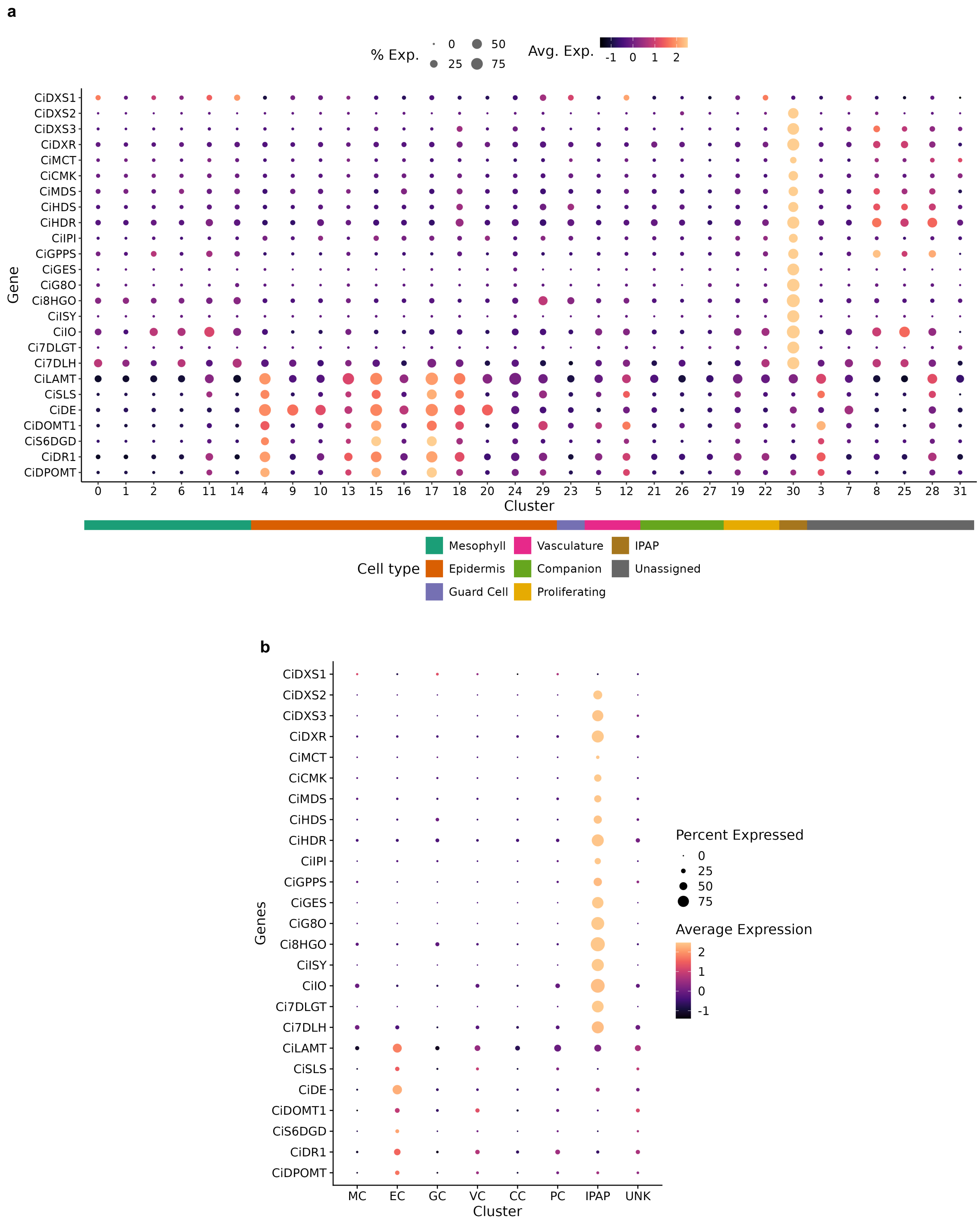

Supplementary Fig. 5. Expression of iridoid and ipecac alkaloid pathway genes in single cell clusters. The expression of orthologs of previously identified 2-C-methyl-D-erythritol 4-phosphate (MEP) pathway, secoiridoid pathway and downstream ipecac alkaloid pathway genes is shown [2,4]. Color scale shows the average scaled expression of each gene over the different cell clusters. Dot sizes indicate the fraction of cells of each cell cluster in which a given gene is expressed. **a**, Expression over 31 cell clusters. **b**, Expression over cell groups of identified cell types. MC, mesophyll cells; EC, epidermal cells; GC, guard cells; VC, vascular cells; CC, companion cells; PC, proliferating cells; IPAP, Internal Phloem Associated Parenchyma; UNK, unassigned.

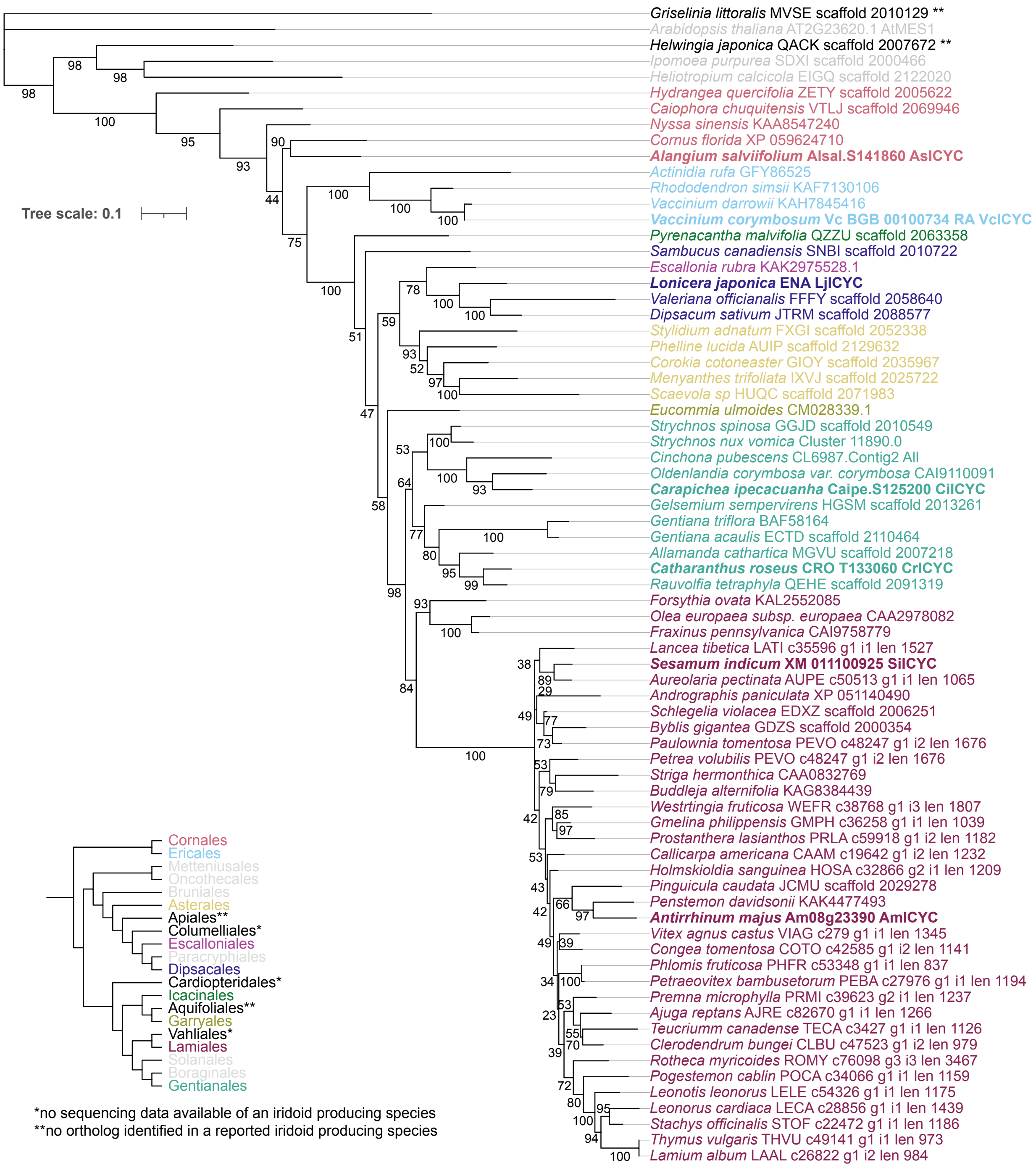

Supplementary Fig. 6. Maximum-likelihood phylogenetic tree of ICYC amino acid sequences. ICYC orthologs were identified in different asterid orders as indicated by the color code. AtMES1 as well the closest ICYC homologs from non-iridoid producing species were included for comparison and do not cluster with ICYCs. In the bottom left corner a tree showing all asterid orders is shown [5]. An asterisk depicts that no sequencing data was publicly available from a reported iridoid producing species from the respective clade and thus ICYC presence could not be determined. Two asterisks depict that no ortholog could be found in the publicly available sequencing data of a reported iridoid producing genus. Orthologs shown in bold were chosen for experimental verification of activities.

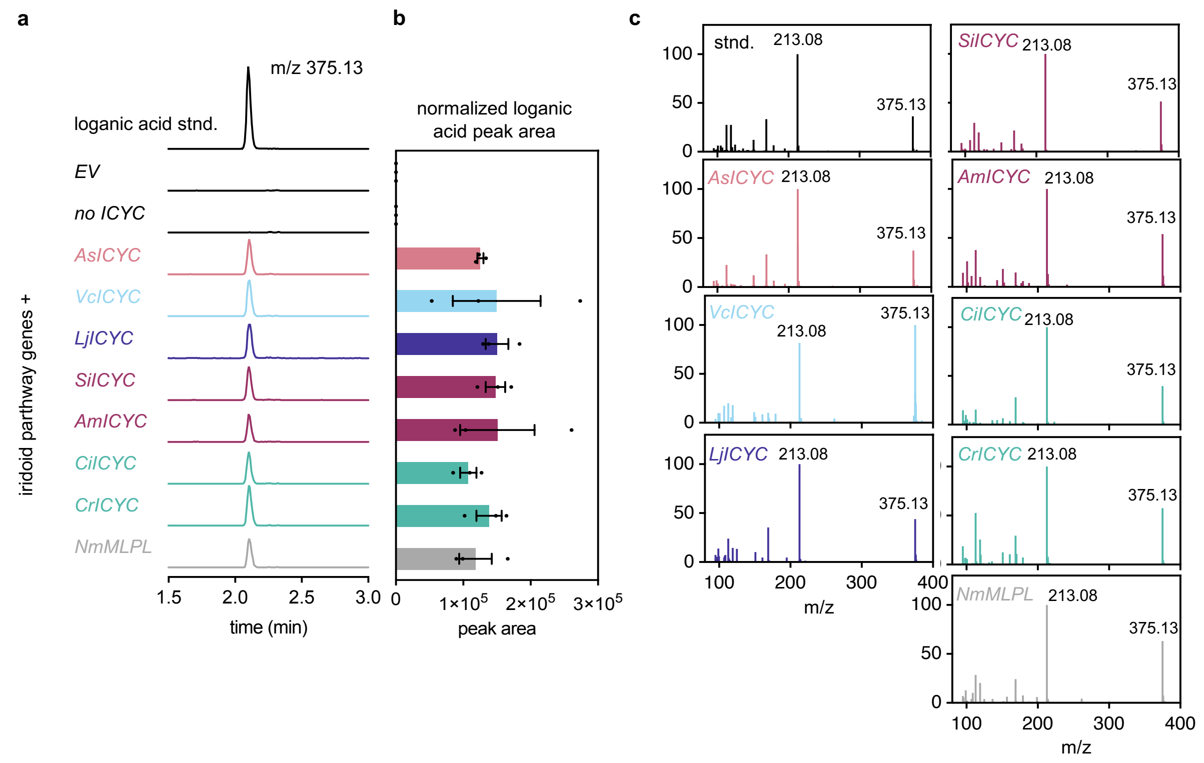

Supplementary Fig. 7. ICYC orthologs from various Asterid orders enable loganic acid biosynthesis in *N. benthamiana*. Full data corresponding to the experiment shown in Fig. 1e (main text). *C. ipecacuanha* loganic acid biosynthesis genes (see Fig. 1e for full list of genes) were co-overexpressed alongside *ICYC* orthologs. **a**, Extracted ion chromatograms (EICs) showing loganic acid *m/z* [M-H]$^-$ 375.13 of a representative biological replicate. **b**, LC-MS peak areas of loganic acid are shown as bars of the mean of N=3 biological replicates, error bars are standard error of the mean. **c**, MS^2 fragmentation data for loganic acid peaks of different samples confirming peaks are identical to the commercial loganic acid standard. Stnd, standard.

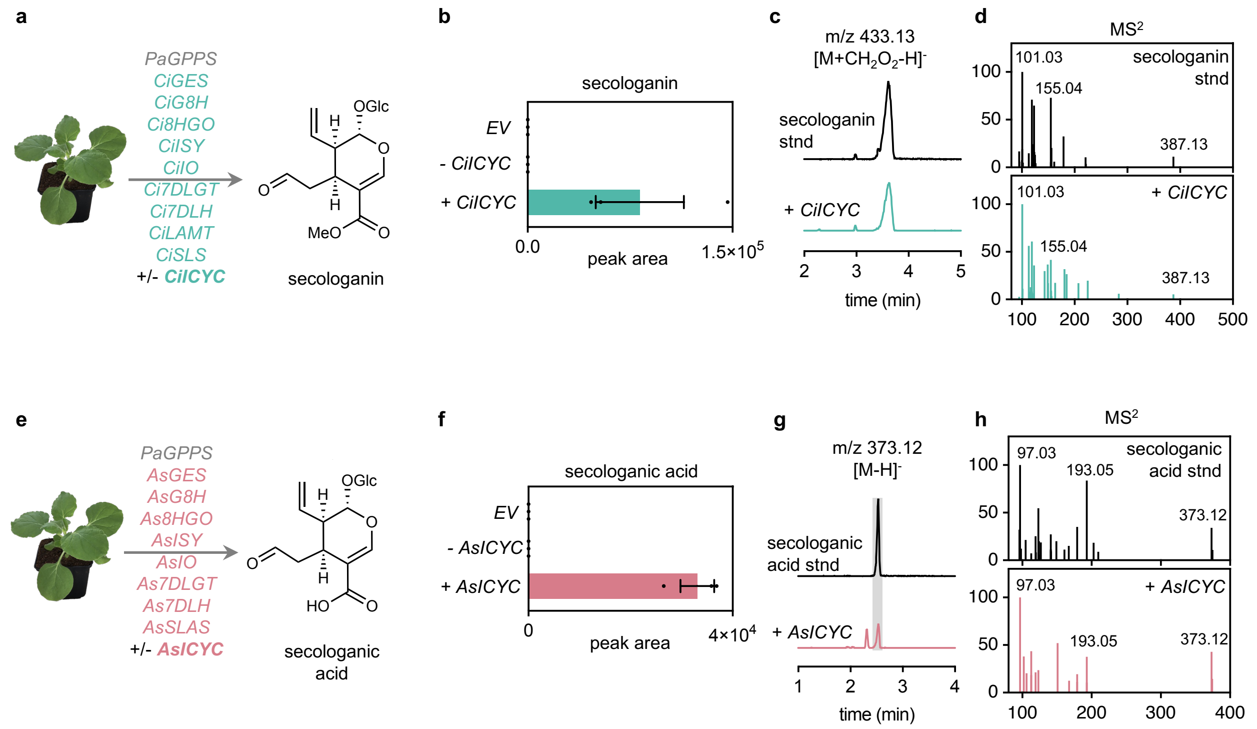

Supplementary Fig. 8. Reconstitution of the secoiridoid pathway of *C. ipecacuanha* and *A. salviifolium* in *N. benthamiana*. a-d, *C. ipecacuanha*. **e-h**, *A. salviifolium*. **a**, **e**, Genes overexpressed through agroinfiltration and expected products. **b**, **f**, Normalized peak areas for indicated products. LC-MS peak areas are shown as bars of the mean of N=3 biological replicates, error bars are standard error of the mean. **c**, **g**, Extracted ion chromatograms alongside authentic standard. **d**, **h** MS^2 data of reconstitution product and standard confirming identity. EV, empty vector; *PaGPPS, Picea abies geraniol diphosphate synthase*; *GES, geraniol synthase; G8H, geraniol 8-hydroxylase; 8HGO, 8-hydroxygeraniol oxidase; ISY, iridoid synthase; ICYC, iridoid cyclase; IO, iridoid oxidase; 7DLGT, 7-deoxyloganetic acid glucosidase; 7DLH, 7-deoxyloganic acid hydroxylase; SLAS, secologanic acid synthase; LAMT, loganic acid methyltransferase; SLS, secologanin synthase.*

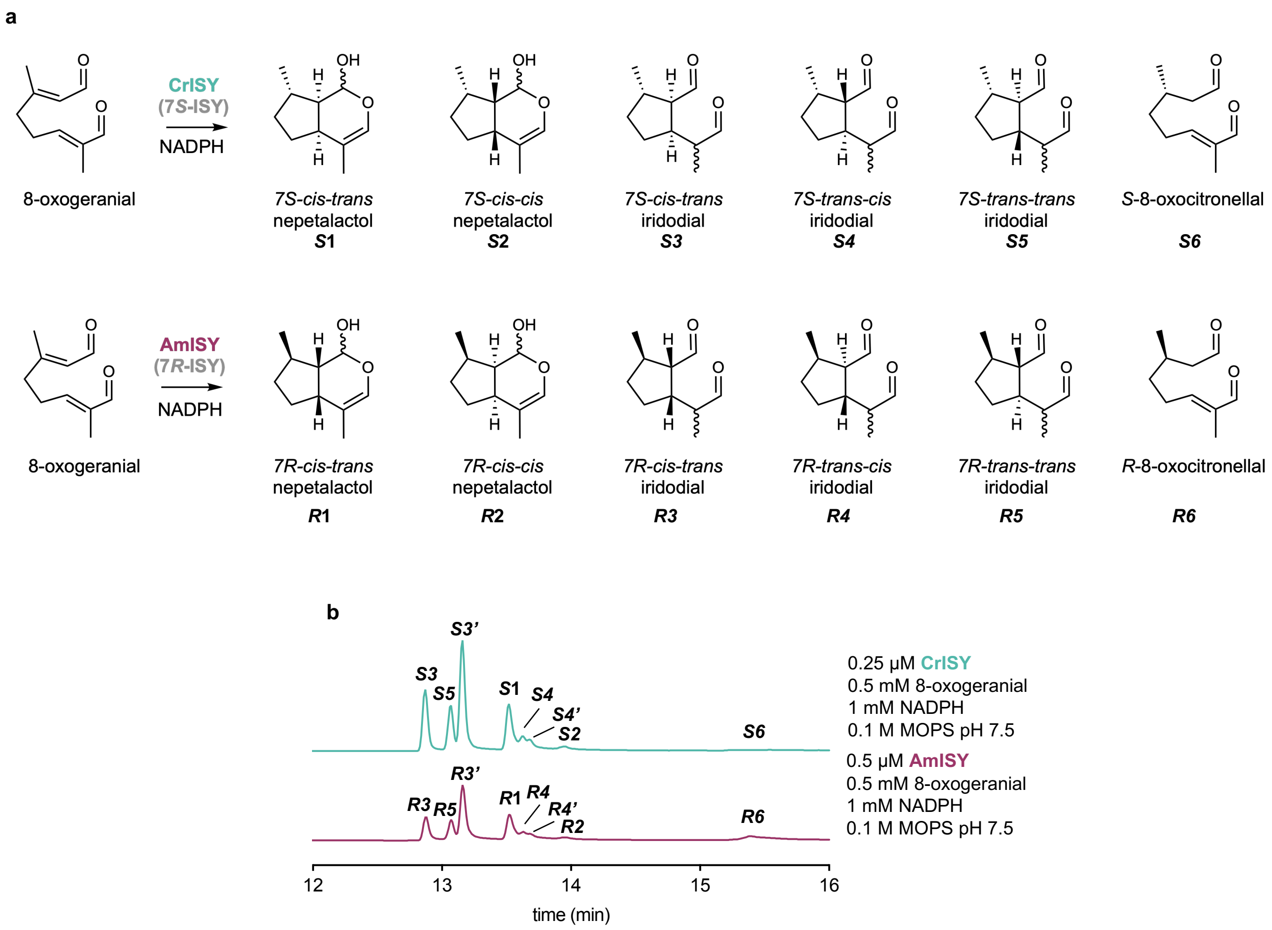

Supplementary Fig. 9. Side products formed by CrISY and AmISY in the absence of cyclase. **a**, Product structures formed by *C. roseus* iridoid synthase (CrISY). **b**, Product structures formed by *Antirrhinum majus* ISY (AmISY). **c**, GC-MS total ion chromatograms (TICs) of ISY catalyzed reactions as indicated. Note that enantiomers co-elute on an achiral column used here. Chromatograms of products were consistent with previously reported work and peak identification was inferred from the previously published profiles [6,7].

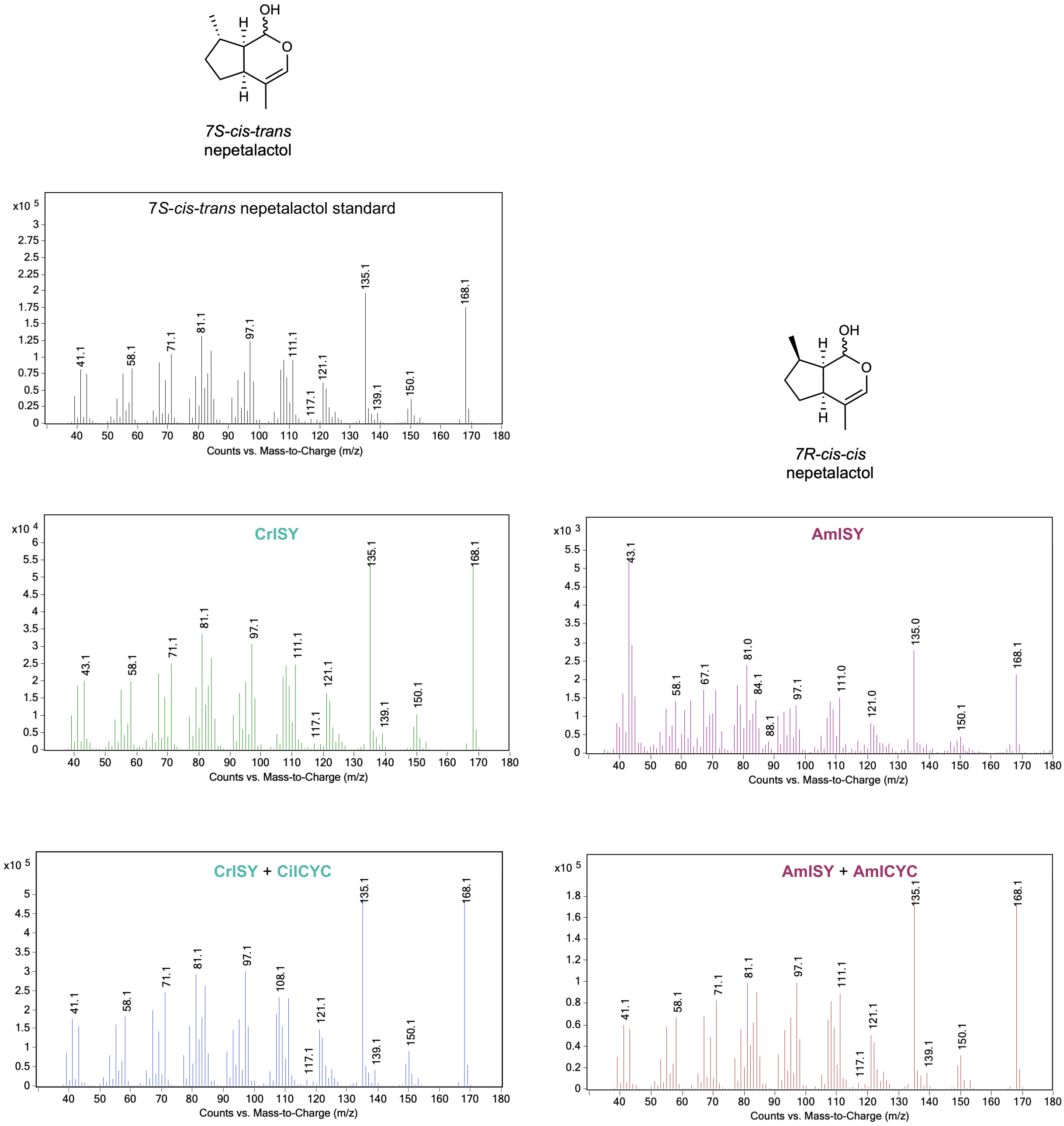

Supplementary Fig. 10. Electron ionization (EI) spectra of nepetalactol standard and enzymatic products. Spectra were obtained from the center of the peaks of chromatograms shown in Fig. 2 b, c.

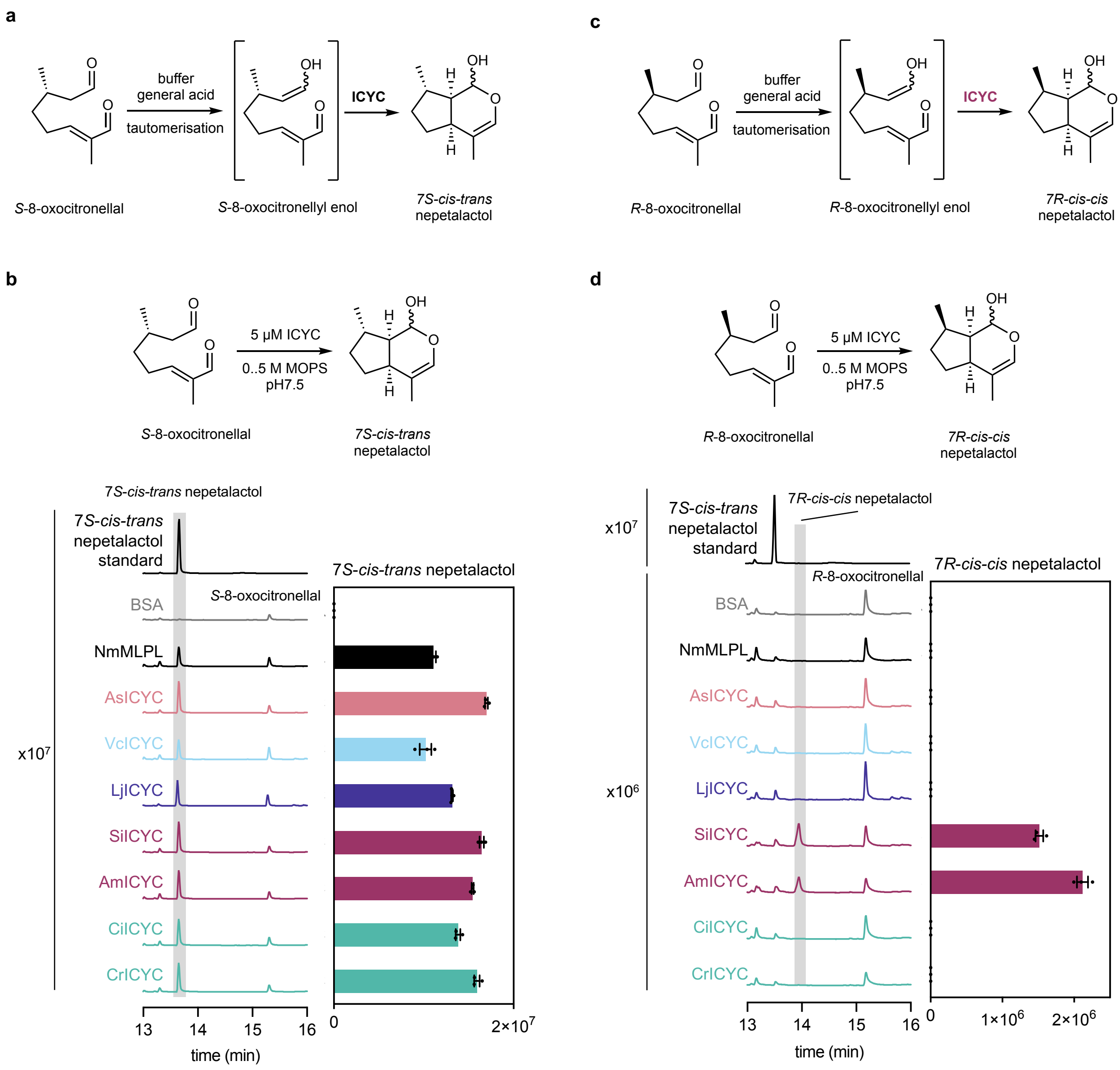

Supplementary Fig. 11. ICYC activity with 8-oxocitronellal under tautomerization inducing conditions. a, **c**, Under high general acid concentrations (0.5 M MOPS, pH 7.5) *S*-8-oxocitronellal (**a**) or *R*-8-oxocitronellal (**c**) partially tautomerize to the cyclase substrate 8-oxocitronellyl enol [6]. **b**, Assays with *S*-8-oxocitronellal (0.5 mM) and ICYC or NmMLPL, or BSA as negative control for 16 hours. 7*S*-*cis*-*trans* nepetalactol was formed in similar amounts by all cyclases but not in the negative control **d**, Assays with *R*-8-oxocitronellal (0.1 mM due to limited substrate availability) for 16 hours. A peak identified as 7*R*-*cis*-*cis* nepetalactol (see Fig. 2d-f, main text) formed specifically in the presence of SiICYC and AmICYC but not with other ICYC orthologs. The results are consistent with the results observed for ISY-ICYC combined assays. LC-MS peak areas are shown as bars of the mean of N=3 replicates, error bars are standard error of the mean.

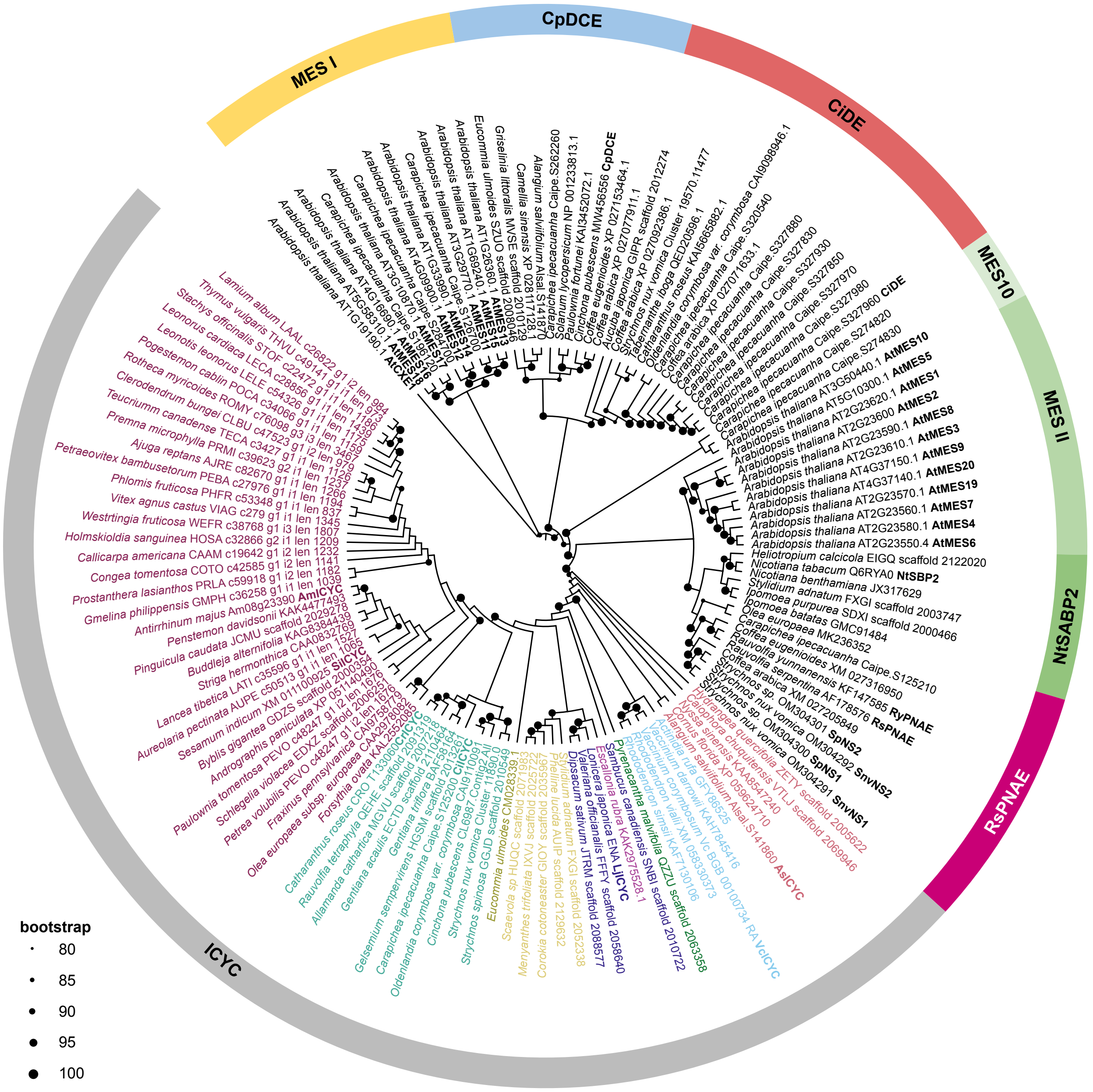

Supplementary Fig. 12. Maximum likelihood tree of ICYC and methylesterase amino acid sequences. *Arabidopsis thaliana* methylesterases (AtMES) were included as well as all *C. ipecacuanha* MES homologs for comparison. *A. thaliana* Carboxylesterase 1 (AtCXE)1 served as outgroup. *Nicotiana tabacum* salicylic acid binding protein 2 (SABP2) has been described as a homolog of AtMES' [8]. MES form distinct clades named I and II whereas MES10-like form a small separate clade. Different alkaloid esterases are found in well separated clades, each clade is named after a characterized member: *Cinchona pubescens* dihydrocorynantheine aldehyde esterase (CpDCE), *Carapichea ipecacuanha* deacetyl(iso)ipecoside esterase (CiDE), *Rauvolfia serpentina* polyneuridine aldehyde esterase (RsPNAE). Enzymes characterized in this or other studies are labelled with names in bold [2,9-12]. NS, norfluorocurarine synthase.

a b

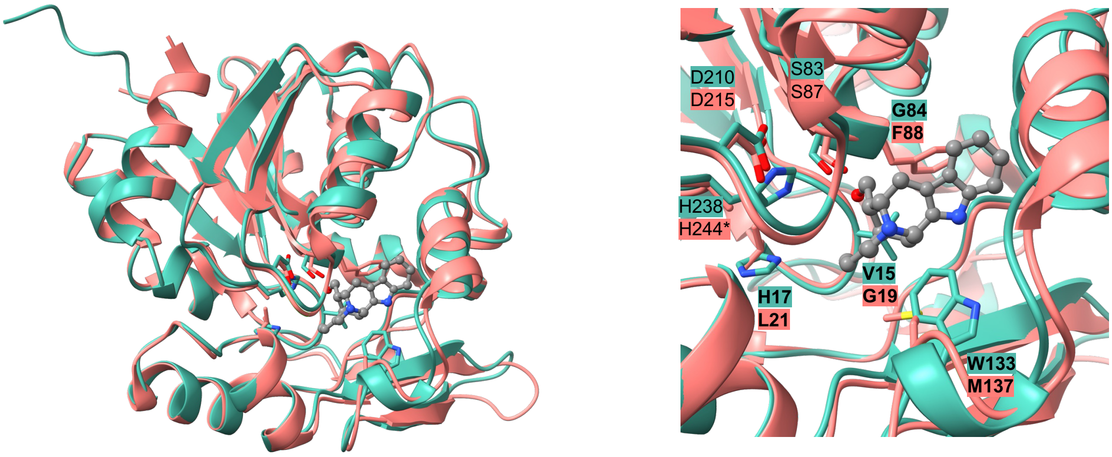

Supplementary Fig. 13. Structural overlay of CiICYC model with RsPNAE structure. Alphafold3 model of CiICYC (in green) was aligned with the crystal structure obtained for *Rauvolfia serpentina* polyneuridine aldehyde esterase (RsPNAE, in salmon) in complex with its product 16-epivellosimine (in grey) (PDB 3GZJ) [13]. RsPNAE is the closest homolog of ICYC for which a crystal structure is available. **a**, Overview of structural alignment showing same protein folds. **b**, Closeup view of the active site. Amino acid residues highlighted in green and shown as sticks were chosen for mutation in CiICYC, corresponding amino acid residues in RsPNAE are also highlighted. In bold, amino acid residues that differed between the two proteins. Asterisk indicates that the native RsPNAE H244 was mutated to alanine in this protein crystal structure.

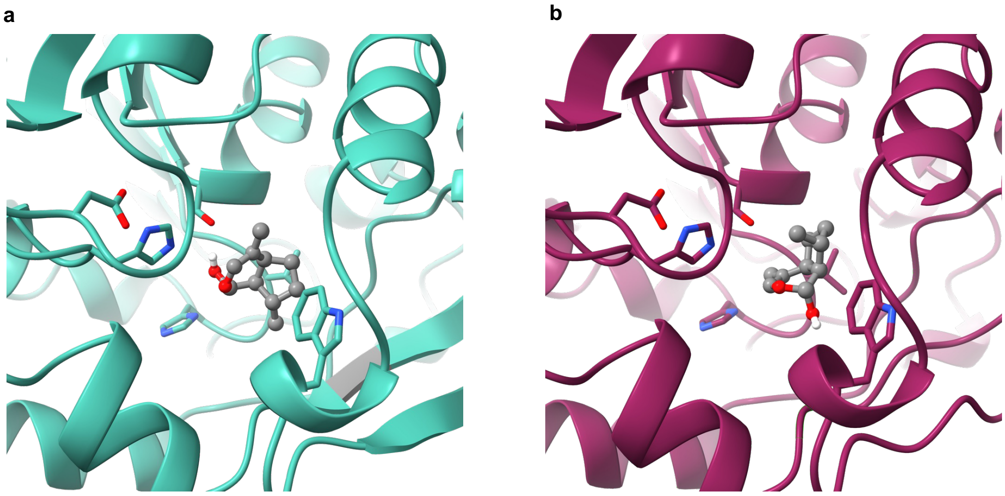

Supplementary Fig. 14. Docking of nepetalactol stereoisomers to CiICYC and AmICYC models. **a**, CiICYC alphafold3 model with 7*S*-*cis*-*trans* nepetalactol docked (AutoDock Vina) in active site. **b**, AmICYC alphafold3 model docked with 7*R*-*cis*-*cis* nepetalactol (Autodock Vina). Amino acid residues shown as sticks were targeted in CiICYC for mutation (Fig 3, main text) and are conserved in all ICYCs.

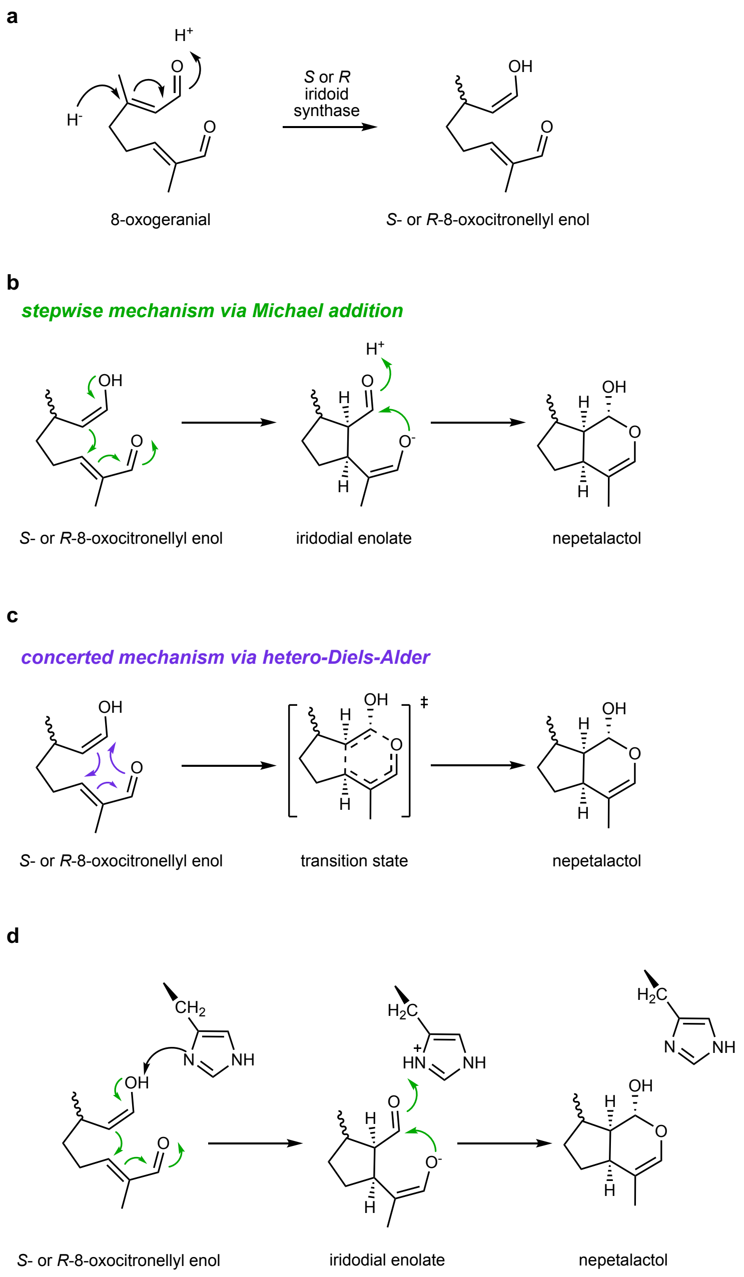

Supplementary Fig. 15. Possible cyclization mechanisms. a, 7*S*-iridoid synthase generates *S*-8-oxocitronellyl enol intermediate. **b**, Stepwise cyclization mechanism via Michael addition occurs via a five-membered ring intermediate. Previous studies using substrate analogs suggest that the spontaneous cyclization occurs via a stepwise Michael addition reaction mechanism [14]. **c**, A possible alternative mechanism is a concerted cyclization via hetero-Diels-Alder. **d**, Proposed role of the active site histidine (H238 in CiICYC) in a putative Michael addition type mechanism.

Supplementary references

1. Kang, M. et al. A chromosome-level Camptotheca acuminata genome assembly provides insights into the evolutionary origin of camptothecin biosynthesis. *Nat Commun* **12**, 3531 (2021).
2. Colinas, M. et al. Ipecac alkaloid biosynthesis in two evolutionarily distant plants. *Nat Chem Biol* (2025).
3. Miller, J.C., Hollatz, A.J. & Schuler, M.A. P450 variations bifurcate the early terpene indole alkaloid pathway in Catharanthus roseus and Camptotheca acuminata. *Phytochemistry* **183**, 112626 (2021).
4. Li, C. et al. Single-cell multi-omics in the medicinal plant Catharanthus roseus. *Nat Chem Biol* **19**, 1031-1041 (2023).
5. Zuntini, A.R. et al. Phylogenomics and the rise of the angiosperms. *Nature* (2024).
6. Lichman, B.R. et al. Uncoupled activation and cyclization in catmint reductive terpenoid biosynthesis. *Nat Chem Biol* **15**, 71-79 (2019).
7. Kries, H., Kellner, F., Kamileen, M.O. & O'Connor, S.E. Inverted stereocontrol of iridoid synthase in snapdragon. *J Biol Chem* **292**, 14659-14667 (2017).
8. Vlot, A.C. et al. Identification of likely orthologs of tobacco salicylic acid-binding protein 2 and their role in systemic acquired resistance in Arabidopsis thaliana. *Plant J* **56**, 445-56 (2008).
9. Trenti, F. et al. Early and Late Steps of Quinine Biosynthesis. *Org Lett* **23**, 1793-1797 (2021).
10. Hong, B. et al. Biosynthesis of strychnine. *Nature* **607**, 617-622 (2022).
11. Dogru, E. et al. The gene encoding polyneuridine aldehyde esterase of monoterpenoid indole alkaloid biosynthesis in plants is an ortholog of the alpha/betahydrolase super family. *Eur J Biochem* **267**, 1397-406 (2000).
12. Chaffin, T.A., Wang, W., Chen, J.-G. & Chen, F. Function and Evolution of the Plant MES Family of Methylesterases. *Plants* **13**(2024).
13. Yang, L., Hill, M., Wang, M., Panjikar, S. & Stockigt, J. Structural basis and enzymatic mechanism of the biosynthesis of C9- from C10-monoterpenoid indole alkaloids. *Angew Chem Int Ed Engl* **48**, 5211-3 (2009).
14. Lindner, S., Geu-Flores, F., Brase, S., Sherden, N.H. & O'Connor, S.E. Conversion of substrate analogs suggests a Michael cyclization in iridoid biosynthesis. *Chem Biol* **21**, 1452-6 (2014).
